# Core Muscle Weakness May Be a Risk Factor for the Development of Plantar Fasciitis

**DOI:** 10.7759/cureus.94954

**Published:** 2025-10-19

**Authors:** Stewart A Bryant, Daniel L Brinton, Griffin J Salzer, Leah N Herzog, Kit N Simpson, Harris S Slone, Shane K Woolf

**Affiliations:** 1 Department of Orthopaedic Surgery, Stanford Health Care, Palo Alto, USA; 2 College of Health Professions, Medical University of South Carolina, Charleston, USA; 3 Department of Emergency Medicine, Beaufort Memorial Hospital, Beaufort, USA; 4 Department of Hand Surgery, Hand Center of Nevada, Las Vegas, USA; 5 Department of Orthopaedics, Medical University of South Carolina, Charleston, USA; 6 Department of Sports Medicine, Sports Medicine &amp; Orthopaedic Centers, Mt. Pleasant, USA

**Keywords:** cesarean section, core weakness, marketscan, plantar fasciitis, plantar fasciosis, posterior spinal fixation and fusion

## Abstract

Introduction

Plantar fasciitis (PF) is a common diagnosis in the United States and worldwide. Core musculature helps to stabilize the spine in order to prevent buckling and provide optimal production of motion. Core deficits may contribute to lower extremity injury through kinematic changes in lower extremity movement. There may be an association between a theoretical core muscle deficit and the subsequent development of symptomatic PF.

Methods

A retrospective case-control study was designed using the Truven Health MarketScan^®^ database. The incidence of new, concurrent PF diagnoses was determined within one year of the date of specific surgeries (cesarean section and posterior lumbar fusion) and for matched controls. The control group was selected using age, sex, employment, region, insurance, Charlson scores, and/or Elixhauser conditions as variables. The adjusted odds of a new PF diagnosis were determined using logistic regression.

Results

Women who underwent cesarean section had 24.1% greater odds of PF within 12 months of delivery compared to vaginal births (odds ratio (OR) 1.241, 95% confidence interval (CI): 1.152-1.336). Patients who underwent posterior lumbar fusion surgery had 11.7% greater odds of developing PF (OR 1.117, 95% CI: 1.084-1.150) than the control group who did not have an existing core or spinal condition diagnosis.

Conclusion

The risk of developing PF is increased after procedures that might create transient core weakness. Given the results of this model, attention to core strengthening and rehabilitation may be of value in the treatment and prevention of PF.

## Introduction

Plantar fasciitis or fasciosis (PF), defined as inflammation at the insertion of the PF, is estimated to affect up to 10% of individuals and accounts for approximately one million provider visits each year in the United States [1,2]. The prevalence of PF has been shown to be higher in individuals who are female, obese (body mass index (BMI) > 30), aged 45-64, or avid runners, or who spend extended time on their feet [2-5]. Despite the prevalence and well-defined risk factors for developing PF, the etiology remains largely unknown [6]. PF can significantly limit a patient’s ability to work, play sports, and attend to daily activities due to pain.

Aside from the demographic risk factors, limited ankle dorsiflexion and gastrocnemius tightness have been found to predict excessive pronation and increase the risk of developing PF [7-9]. Other anatomical risk factors for experiencing PF include pes planus, pes cavus, increased subtalar pronation, lateral tibial torsion, varus alignment of the knee or hindfoot, and increased femoral anteversion [4,10,11]. While many anatomic and muscular risk factors have been reported, limited literature on core stability as a risk factor of PF exists.

The definitions of core and core stability have been debated in the literature [12-14]. The core can be described in many ways, but most include muscles of the lumbo-pelvic-hip complex as well as the abdominal and proximal lower extremity musculature [13,15]. Core stability has become a more nebulous concept and, for simplicity, may be considered as the strength and control of the core to stabilize the spine in order to maintain posture, prevent buckling, and provide optimal production of motion [12-15]. Current research on core strengthening suggests a decreased risk of lower extremity injury [15,16]. Additionally, clinical guidelines have referenced hip abductor weakness as a mild risk factor for PF [17]. Research recommends the use of lower-body strengthening for the treatment of PF [18]. However, research specifically addressing core strength in relation to PF is lacking. The purpose of this study is to assess if an association exists between core weakness and the development of PF. Our hypothesis is that there is an association between core muscle deficit and the subsequent development of symptomatic PF.

## Materials and methods

Study design

This study is a retrospective analysis of archival billing data from Truven Health MarketScan^®^. The Truven MarketScan^®^ Commercial Claims database is a US-based insurance claims database for all areas of clinical practice, from office visits to hospital stays. It contains over 50 million deidentified patient records including age, sex, geographic region, insurance plan, and employment status in addition to the International Classification of Diseases, 9th Revision (ICD-9) and Current Procedure Terminology, 4th edition (CPT) codes filed for each patient [19,20]. Subjects in the database are at least 18 years old.

MarketScan^®^ data were extracted using selected ICD-9 codes for the index surgery claim during the year 2013 and baseline data from 2012. Our model used procedures that could be expected to weaken core strength by affecting specific structural components of the core to create surrogates for a prospective, transient core deficit. The incidence of concurrent PF diagnoses documented within one year of the date of the core-related procedure was determined using these data. The primary outcome was the development of PF within one year of the core-insulting procedure. The diagnosis of PF was determined by the presence of the ICD-9 code 728.71. The incidence of PF was considered a secondary outcome.

Procedures that could weaken core strength by affecting specific structural components of the core were used to create the surrogates for the transient core deficit. The control groups were propensity score-matched using baseline characteristics from the three months prior to surgery using a Greedy algorithm using SAS version 9.4 (SAS Inc., Cary, NC, US). Matching variables included age, Charlson score, sex (when appropriate), employment status, geographic region, health plan type, and 27 Elixhauser conditions (when appropriate). The Charlson scores were constructed from inpatient records, while the Elixhauser score was based on outpatient diagnosis codes [21,22]. These scores allowed for propensity matching based on comorbidities noted in both inpatient and outpatient settings. The study sample size was determined by the available data for each model in the database after matching.

Inclusion and exclusion criteria

Two models were matched as the experimental and control groups. Patients were included if they had either the corresponding ICD or CPT codes for the specific model as outlined in the respective sections below. Table 1 outlines the ICD and CPT codes used in the design of the specific groups. Patients under the age of 18 were excluded from the database. Additionally, patients with a diagnosis code of BMI > 30, morbid obesity (ICD-9 278.00, 278.01), or a previous diagnosis of PF were excluded from all surgery and control datasets.

**Table 1:** ICD-9 and CPT codes by groups with associated descriptions ICD: International Classification of Diseases; CPT: Current Procedural Terminology; CS: cesarean (C) section; SVD: spontaneous vaginal delivery; PLF: posterior lumbar fusion; URI: upper respiratory infection Source: References [19,20]

	ICD-9 (CPT code)	Description
CS	74.0; 74.1; 74.2; (59510); (59515); (59525)	Classical cesarean section; Low cervical cesarean section; Extraperitoneal cesarean section; C-section delivery including antepartum and postpartum care; C-section delivery including postpartum care; Subtotal or total hysterectomy after cesarean delivery
SVD	650	Normal delivery
PLF	721.3; 721.42; 722.52; 724.02; (8105); (8107); (8108)	Lumbosacral spondylosis without myelopathy; Spondylosis with myelopathy, lumbar region; Degeneration of lumbar or lumbosacral intervertebral disc; Spinal stenosis, lumbar region, without neurogenic claudication; Dorsal and dorsolumbar fusion of posterior column, posterior technique; Lumbar and lumbosacral fusion of posterior column, posterior technique; Lumbar and lumbosacral fusion of anterior column, posterior technique
URI	465.9	Acute upper respiratory infections of unspecified site

Cesarean Section and Spontaneous Vaginal Delivery

Cesarean section (CS) was considered a model for transient insult to the core because of its lower abdominal incision and separation of the rectus sheath and rectus abdominus as well as postpartum restrictions, which rest the abdominal musculature. Spontaneous vaginal delivery (SVD) was selected as a natural control group for the CS group because it was similar in duration and changes in the body but lacked the transient core deficit seen with CS. The CS group was formed using ICD-9 codes (74.0-74.2) and CPT (59510, 59515, and 59525). The SVD group was built using the ICD-9 code 650.xx. Elixhauser scores were not used in the matching process because of a few comorbidities within the groups.

Posterior Lumbar Fusion and Upper Respiratory Infection

Posterior lumbar fusion (PLF) was considered a model for transient insult to the core because of its low back incision and manipulation of the erector spinae muscles. Upper respiratory infections (URIs) without low back pain or other core deficit conditions were selected as a control group for the PLF group because it was considered to have a low likelihood of core deficit. Controls were matched in a 2-1 ratio, given the availability of control cases and the ability to improve statistical power. Exclusion criteria for the PLF cohort were previous spinal surgery. Exclusion criteria for the URI group included back injury, previous core insulting surgery, insurance less than 12 months before and after the surgery or diagnosis, or inguinal hernia. The PLF group was specified using ICD-9 codes 721.3, 721.42, 722.52, and 724.02 and CPT codes 8105, 8107, and 8108. The URI group was built using the ICD-9 code 465.9.

Statistical analysis

Comparisons between the matched groups were performed using descriptive statistics for patients undergoing a procedure and the control. Comparison of baseline demographics pre- and post-propensity score matching was performed using Student’s t-test or Mann-Whitney U for continuous variables and the Chi-squared test for categorical variables.

The adjusted odds of PF diagnosis within one year of surgical intervention were estimated using logistic regression. The logistic regression controlled for age, sex (where appropriate), and comorbid conditions based on the Charlson comorbidity index. All analyses were performed using SAS version 9.4 (SAS Inc., Cary, NC, US). Statistical significance was defined as p < 0.05.

## Results

Matching

**Figure 1:**
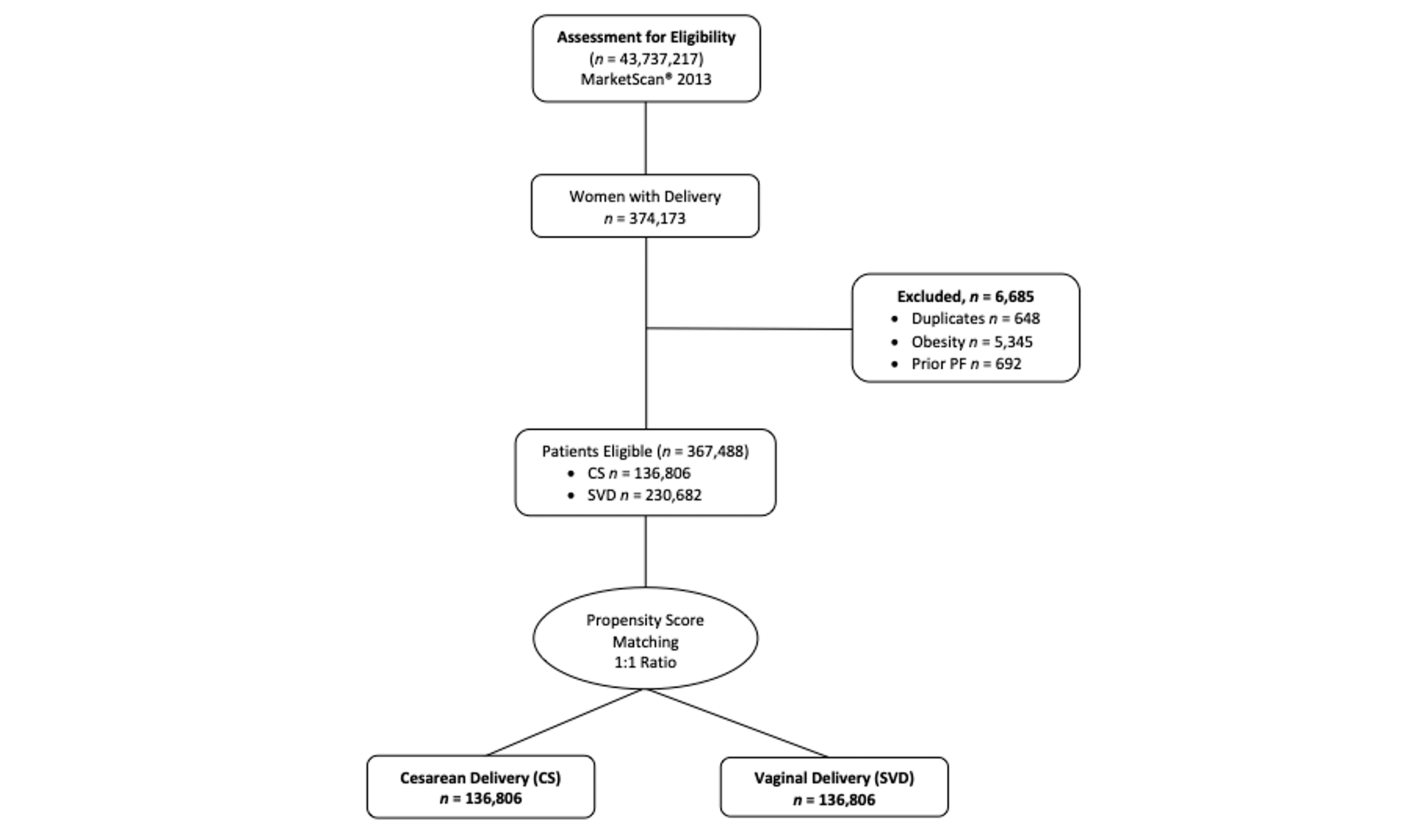
Flow diagram of selection criteria for the CS and SVD groups PF: plantar fasciitis; CS: cesarean section; SVD: spontaneous vaginal delivery

The CS and SVD groups were matched using propensity scoring from the Charlson data, age, and exclusion criteria. Figure 1 shows a flow diagram for the participant matching of the CS and SVD groups.

The PLF and URI groups were matched using the propensity scoring from the Charlson and Elixhauser data. The results for the matching of the PLF and URI groups are in Table 2 and include selected Elixhauser data.

**Table 2:** Propensity matching statistics for PLF and URI groups including age, sex, and selected Elixhauser scores n: number; SD: standard deviation; %: percentage; COPD: chronic obstructive pulmonary disease

Matching characteristic	Upper respiratory infection (URI)	Posterior lumbar fusion (PLF)
n	730,042	365,021
Age of patient (±SD)	48.9 (±11.9)	48.6 (±11.8)
Male (%)	341,732 (46.8%)	170,265 (46.6%)
Charlson score (±SD)	0.0 (±0.3)	0.1 (±0.6)
Elixhauser: alcohol abuse (%)	7,620 (1.0%)	4,442 (1.2%)
Elixhauser: blood loss anemia (%)	2,092 (0.3%)	1,226 (0.3%)
Elixhauser: COPD (%)	74,218 (10.2%)	38,787 (10.6%)
Elixhauser: congestive heart failure (%)	8,276 (1.1%)	5,030 (1.4%)
Elixhauser: diabetes, uncomplicated (%)	87,036 (11.9%)	44,161 (12.1%)
Elixhauser: hypertension, uncomplicated (%)	235,074 (32.2%)	118,515 (32.5%)
Elixhauser: peptic ulcer disease, excludes bleeding (%)	3,277 (0.4%)	2,031 (0.6%)
Elixhauser: peripheral vascular disorder (%)	16,864 (2.3%)	10,063 (2.8%)
Elixhauser: renal failure (%)	11,551 (1.6%)	7,087 (1.9%)
Elixhauser: rheumatoid arthritis (%)	49,214 (6.7%)	27,137 (7.4%)

**Figure 2:**
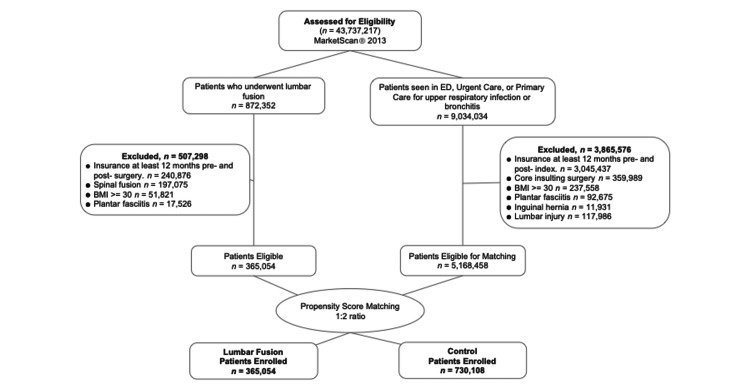
Flow diagram of selection criteria for posterior lumbar fusion and upper respiratory infection groups BMI: body mass index

Figure 2 shows a flow diagram for the participant matching of the PLF and URI groups.

Baseline characteristics

The CS cohort had 136,806 patients in the CS group and 136,806 in the control (SVD). The PLF cohort had 365,054 patients in the PLF group and 730,108 in the control (URI). The number of patients that developed PF in all groups over the course of one year was 22,371 for an overall incidence of 1.63%. Overall incidence of PF in the surgery groups was 8,524 (1.70%). Overall incidence of PF in the control groups was 13,847 (1.60%). Demographic characteristics for the matched groups are shown in Table 3.

**Table 3:** Descriptive statistics by groups including number, age, sex, Charlson score, and incidence of plantar fasciitis CS: cesarean section; SVD: spontaneous vaginal delivery; PLF: posterior lumbar fusion; URI: upper respiratory infection; SD: standard deviation; PF: plantar fasciitis; NA: not applicable * indicated p < 0.05

Group	Number	Age (±SD)	Charlson (±SD)	PF (%)
CS	136,806	31.1 (±5.6)	0.03 (±0.21)	1,583 (1.16%)^*^
SVD	136,806	31.1 (±5.5)	0.03 (±0.19)	1,286 (0.94%)
PLF	365,054	48.6 (±11.8)	0.1 (±0.6)	6,941 (1.9%)^*^
URI	730,108	48.9 (±11.9)	0 (±0.3)	12,561 (1.7%)

Results of models for transient core weakness

**Figure 3:**
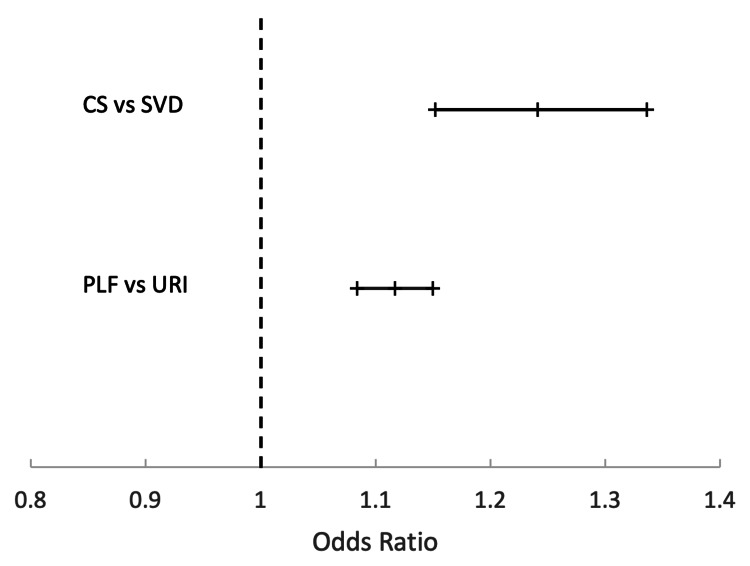
Odds ratio comparison and 95% CI by group CS: cesarean section; SVD: spontaneous vaginal delivery; PLF: posterior lumbar fusion; URI: upper respiratory infection; CI: confidence interval

The CS group had an incidence of PF of 1.16% compared to 0.97% in the SVD group. Women who underwent CS had 24.1% greater odds of developing PF within 12 months after delivery compared to SVD (odds ratio (OR) 1.241, 95% confidence interval (CI): 1.152-1.336). The PLF group had an incidence of PF of 1.9% compared to 1.7% in the URI group. Patients who underwent PLF surgery had 11.7% greater odds of developing PF within 12 months compared to individuals diagnosed with URI (OR 1.117, 95% CI: 1.084-1.150). Figure 3 graphically depicts the OR with 95% CI for the development of PF in each core deficit model.

## Discussion

PF is well studied and has a strong natural history of resolution over the course of one year [1,23]. However, pain and functional limitations due to PF can be impactful. Despite studies concerning treatment, the underlying cause of PF is not well understood [6]. The poor understanding of the cause and impact of PF on physical activity warrants investigation into better therapy and prevention [24]. Further investigation into the causes of PF may better guide therapy. The results of this study demonstrate evidence for an association between transient core strength deficit and the development of PF.

The incidence of PF in this study was 1.63% in one year for all the combined groups, similar to other population surveys [5]. Supporting an established incidence may help establish outlier information within large datasets, such as in this study. Producing an increased incidence at a population level may inform the demographics prone to developing PF. This study emphasized the potential increase in PF after patients underwent core-injuring events and used two examples to demonstrate an increase in incidence within a large, controlled dataset.

The CS and SVD model found that patients who underwent CS were 24.1% more likely to have symptomatic PF the following year after birth. The model design allowed for hormonal, gait, muscle weakness, joint laxity, and pelvic changes to be controlled during the preceding nine months prior to delivery [25-28]. Related studies of plantar pressure and foot pronation have noted changes that occur during pregnancy [29,30]. However, these studies did not comment on PF or the risk associated with these changes. Foti et al. postulated that gait changes in pregnancy may contribute to overuse injuries in the lower extremities [25]. Surveys of women throughout pregnancy noted 65%-75% experienced leg cramping and 36%-37% had foot or ankle pain by the third trimester [31,32]. Though there is a postulated increased likelihood of lower extremity injury, there is a paucity of literature documenting its incidence.

The PLF and URI model found that patients who underwent a PLF surgery were 11.7% more likely to have symptomatic PF the following year. The PLF group was compared to a group without core or back conditions, allowing the data to suggest a relationship between theoretical transient weakness of the core and the development of PF. The PLF and URI model, unlike the CS and SVD model, was different because prior gait, core, or back problems existing before the PLF were not controlled. Given that core musculature provides stability for the lumbar spine, the effect of surgery would potentially influence the development of PF [13]. Back pain itself is considered a risk factor for plantar heel pain. A cross-sectional study found that back pain increased the odds of plantar heel pain by five times [33]. Additionally, the smaller influence of surgery may be due to the balanced genders of the group. Since men are considered at lower risk of developing PF, their presence in the model may have weakened the effect of surgery [2,5].

Similar studies establish a possible link between core weakness and lower extremity injury. Studies have found that increased incidence of patellofemoral pain is associated with decreasing core muscle size and weakness of ipsilateral hip abduction and external rotation [34,35]. Multiple studies have associated the risk of lower extremity injury with hip weakness [15,36-38]. Weakness in core flexion and extension strength has been implicated as a risk factor for anterior cruciate ligament (ACL) injury [39]. Gluteal weakness is linked as a risk factor for ankle sprain [40,41].

A review of hip strength and lower extremity injuries noted a relationship between ankle instability and altered hip biomechanics as well as a gap in the literature related specifically to plantar heel pain [41]. Core deficits may similarly contribute to injury of the lower extremity through changes in hip and knee kinematics. A link between core weakness and the development of PF has not been previously reported. This study is the first to consider a correlation between core deficits and the development of PF and provided data supporting increased odds during transient core deficits.

Current modalities for the treatment of PF include stretching and strength training [6,42,43]. Most studies have focused on foot and ankle-specific training. A randomized trial found that hip training with foot stretching decreases pain from PF; however, there was no significant difference compared to stretching alone [35]. As of this study, two case reports highlighted improvement in heel pain associated with hip strengthening after failure of heel-specific stretching and strengthening and in combination with manipulative therapy [44,45]. Finally, a 30-patient prospective study found that strengthening of the gluteal musculature decreased pain associated with PF [46]. Our study provides evidence that core weakness may have an association with PF, and thus, core strengthening modalities could provide benefit in PF therapy.

This study does have many limitations inherent in any population-based study using diagnostic codes and database information. The methodology of our study permits the establishment of an association between the risk factor for a transient core insult and the development of PF. This association does not suggest causality. Multiple risk factors were not controlled for in the creation of the groups studied. The surgical interventions have not been proven to cause a core weakness, nor has a degree of weakness been determined. The CS group did not have data on the type of CS or whether techniques such as rectus muscle re-approximation were performed during closure. Gestational age < 37 weeks was not identified from the CS or SVD cohorts and may have influenced the results.

The PLF group did not have a nature comparison like the pregnancy group, and the potential for variability related to its URI comparison exists. The PLF and URI groups did not control for pregnancy, which could influence the results, although we anticipate this would be of minimal impact. The PLF and URI groups did not have the same exclusion criteria, which could diminish their comparison. PLF was done for chronic changes in the back, potentially correcting gait and stability abnormalities. This could have blunted the effect of the transient core deficit in the lumbar surgery model. These corrections could mitigate the differences due to the surgery. The extent of the spinal fusion is also not documented and could influence the study results. The models’ use of surgical interventions as a surrogate for core weakness may limit the generalizability to populations that have inherent core weakness or core injuries. However, these models may be more generalizable than, for example, athletics cohorts, given the broad population included and matched design.

## Conclusions

PF may have a relationship with core weakness, given the increased incidence following surgeries in our study model, which theoretically could cause transient core weakness. Based on our findings, further clinical study of rehabilitation of the musculoskeletal core in the cause, prevention, and treatment of PF is warranted. Given the potential for therapeutic benefit from core rehabilitation, a comprehensive treatment plan for PF may include core stability training. These findings may impact rehabilitation potential in PF and consideration for high-risk patients. In addition, our findings indirectly add to evidence that core training may help in avoiding lower extremity injury, particularly in active individuals. Further research on the utility of core strengthening and its impact on the prevention and treatment of PF and other lower extremity injuries is necessary.

## References

[1] Crawford F, Thomson C (2003). Interventions for treating plantar heel pain. Cochrane Database Syst Rev.

[2] Riddle DL, Schappert SM (2004). Volume of ambulatory care visits and patterns of care for patients diagnosed with plantar fasciitis: a national study of medical doctors. Foot Ankle Int.

[3] Lopes AD, Hespanhol Júnior LC, Yeung SS, Costa LO (2012). What are the main running-related musculoskeletal injuries? A systematic review. Sports Med.

[4] Wearing SC, Smeathers JE, Urry SR, Hennig EM, Hills AP (2006). The pathomechanics of plantar fasciitis. Sports Med.

[5] Nahin RL (2018). Prevalence and pharmaceutical treatment of plantar fasciitis in United States adults. J Pain.

[6] Sweeting D, Parish B, Hooper L, Chester R (2011). The effectiveness of manual stretching in the treatment of plantar heel pain: a systematic review. J Foot Ankle Res.

[7] Bolívar YA, Munuera PV, Padillo JP (2013). Relationship between tightness of the posterior muscles of the lower limb and plantar fasciitis. Foot Ankle Int.

[8] Riddle DL, Pulisic M, Pidcoe P, Johnson RE (2003). Risk factors for plantar fasciitis: a matched case-control study. J Bone Joint Surg Am.

[9] van Leeuwen KD, Rogers J, Winzenberg T, van Middelkoop M (2016). Higher body mass index is associated with plantar fasciopathy/'plantar fasciitis': systematic review and meta-analysis of various clinical and imaging risk factors. Br J Sports Med.

[10] Di Caprio F, Buda R, Mosca M, Calabro' A, Giannini S. (2010). Foot and lower limb diseases in runners: assessment of risk factors. J Sports Sci Med.

[11] Pohl MB, Hamill J, Davis IS (2009). Biomechanical and anatomic factors associated with a history of plantar fasciitis in female runners. Clin J Sport Med.

[12] Hibbs AE, Thompson KG, French D, Wrigley A, Spears I (2008). Optimizing performance by improving core stability and core strength. Sports Med.

[13] Kibler WB, Press J, Sciascia A (2006). The role of core stability in athletic function. Sports Med.

[14] Willson JD, Dougherty CP, Ireland ML, Davis IM (2005). Core stability and its relationship to lower extremity function and injury. J Am Acad Orthop Surg.

[15] Leetun DT, Ireland ML, Willson JD, Ballantyne BT, Davis IM (2004). Core stability measures as risk factors for lower extremity injury in athletes. Med Sci Sports Exerc.

[16] De Blaiser C, Roosen P, Willems T, Danneels L, Bossche LV, De Ridder R (2018). Is core stability a risk factor for lower extremity injuries in an athletic population? A systematic review. Phys Ther Sport.

[17] Martin RL, Davenport TE, Reischl SF, McPoil TG, Matheson JW, Wukich DK, McDonough CM (2014). Heel pain-plantar fasciitis: revision 2014. J Orthop Sports Phys Ther.

[18] Lee JH, Jung HW, Jang WY (2020). A prospective study of the muscle strength and reaction time of the quadriceps, hamstring, and gastrocnemius muscles in patients with plantar fasciitis. BMC Musculoskelet Disord.

[19] Centers for Disease Control and Prevention, National Center for Health Statistics, Centers for Medicare & Medicaid Services (2014). International Classification of Diseases, Ninth Revision, Clinical Modification (ICD-9-CM). https://archive.cdc.gov/www_cdc_gov/nchs/icd/icd9cm.htm.

[20] American Medical Association (2014). CPT® 2015 Professional Edition. American Medical Association.

[21] Charlson ME, Pompei P, Ales KL, MacKenzie CR (1987). A new method of classifying prognostic comorbidity in longitudinal studies: development and validation. J Chronic Dis.

[22] Elixhauser A, Steiner C, Harris DR, Coffey RM (1998). Comorbidity measures for use with administrative data. Med Care.

[23] Davis PF, Severud E, Baxter DE (1994). Painful heel syndrome: results of nonoperative treatment. Foot Ankle Int.

[24] Orchard J (2012). Plantar fasciitis. BMJ.

[25] Foti T, Davids JR, Bagley A (2000). A biomechanical analysis of gait during pregnancy. J Bone Joint Surg Am.

[26] Krkeljas Z (2018). Changes in gait and posture as factors of dynamic stability during walking in pregnancy. Hum Mov Sci.

[27] Mota P, Pascoal AG, Carita AI, Bø K (2015). The immediate effects on inter-rectus distance of abdominal crunch and drawing-in exercises during pregnancy and the postpartum period. J Orthop Sports Phys Ther.

[28] Ritchie JR (2003). Orthopedic considerations during pregnancy. Clin Obstet Gynecol.

[29] Karadag-Saygi E, Unlu-Ozkan F, Basgul A (2010). Plantar pressure and foot pain in the last trimester of pregnancy. Foot Ankle Int.

[30] Ramachandra P, Kumar P, Kamath A, Maiya AG (2017). Do structural changes of the foot influence plantar pressure patterns during various stages of pregnancy and postpartum?. Foot Ankle Spec.

[31] Kesikburun S, Güzelküçük Ü, Fidan U, Demir Y, Ergün A, Tan AK (2018). Musculoskeletal pain and symptoms in pregnancy: a descriptive study. Ther Adv Musculoskelet Dis.

[32] Ramachandra P, Maiya AG, Kumar P, Kamath A (2015). Prevalence of musculoskeletal dysfunctions among Indian pregnant women. J Pregnancy.

[33] McClinton S, Weber CF, Heiderscheit B (2018). Low back pain and disability in individuals with plantar heel pain. Foot (Edinb).

[34] Briani RV, Waiteman MC, de Albuquerque CE (2019). Lower trunk muscle thickness is associated with pain in women with patellofemoral pain. J Ultrasound Med.

[35] Kamonseki DH, Gonçalves GA, Yi LC, Júnior IL (2016). Effect of stretching with and without muscle strengthening exercises for the foot and hip in patients with plantar fasciitis: a randomized controlled single-blind clinical trial. Man Ther.

[36] Abdallah AA, Mohamed NA, Hegazy MA (2017). A comparative study of core musculature endurance and strength between soccer players with and without lower extremity sprain and strain injury. Osteoarthr Cartil.

[37] Ireland ML, Willson JD, Ballantyne BT, Davis IM (2003). Hip strength in females with and without patellofemoral pain. J Orthop Sports Phys Ther.

[38] Nadler SF, Malanga GA, DePrince M, Stitik TP, Feinberg JH (2000). The relationship between lower extremity injury, low back pain, and hip muscle strength in male and female collegiate athletes. Clin J Sport Med.

[39] Raschner C, Platzer HP, Patterson C, Werner I, Huber R, Hildebrandt C (2012). The relationship between ACL injuries and physical fitness in young competitive ski racers: a 10-year longitudinal study. Br J Sports Med.

[40] Beckman SM, Buchanan TS (1995). Ankle inversion injury and hypermobility: effect on hip and ankle muscle electromyography onset latency. Arch Phys Med Rehabil.

[41] Steinberg N, Dar G, Dunlop M, Gaida JE (2017). The relationship of hip muscle performance to leg, ankle and foot injuries: a systematic review. Phys Sportsmed.

[42] Digiovanni BF, Nawoczenski DA, Malay DP, Graci PA, Williams TT, Wilding GE, Baumhauer JF (2006). Plantar fascia-specific stretching exercise improves outcomes in patients with chronic plantar fasciitis. A prospective clinical trial with two-year follow-up. J Bone Joint Surg Am.

[43] Rathleff MS, Mølgaard CM, Fredberg U (2015). High-load strength training improves outcome in patients with plantar fasciitis: a randomized controlled trial with 12-month follow-up. Scand J Med Sci Sports.

[44] Lee JH, Park JH, Jang WY (2019). The effects of hip strengthening exercises in a patient with plantar fasciitis: a case report. Medicine (Baltimore).

[45] Santos BD, Corrêa LA, Teixeira Santos L, Filho NA, Lemos T, Nogueira LA (2016). Combination of hip strengthening and manipulative therapy for the treatment of plantar fasciitis: a case report. J Chiropr Med.

[46] John J, Augustine J (2020). Effectiveness of gluteal muscles strengthening in patients with plantar fasciitis. International Journal Medical and Exercise Science.

